# Correction to Exosomal circDNER enhances paclitaxel resistance and tumorigenicity of lung cancer via targeting miR‐139‐5p/ITGB8

**DOI:** 10.1111/1759-7714.14857

**Published:** 2023-03-22

In Jinyou Li et al.^1^ the following errors were published on page 1384.

The exosome identification result of A549 cell line in Figure 2a was misused which was the 80 000x result of A549/PTX cell line (Figure B). The correct one should be the A549 cell line 100 000x exosome identification result (Figure A).

FIGURE A Correct A549 cell line exosome electron microscope result(100 000x).

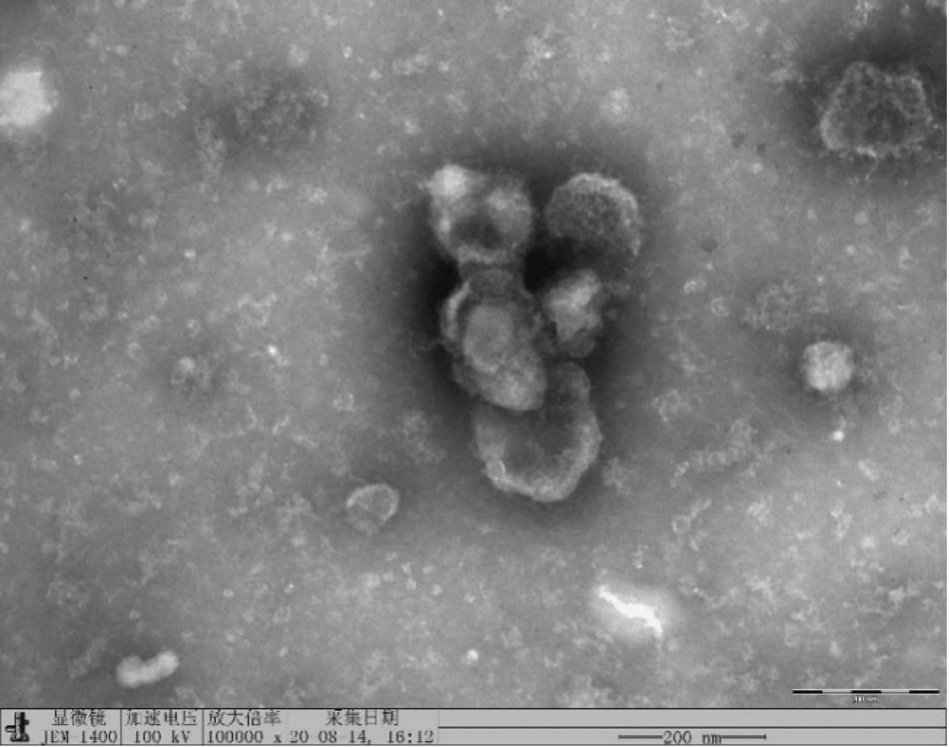

FIGURE B The article is now regarded as the electron microscopy results of the A549/PTX cell line misused by A549(80 000x).

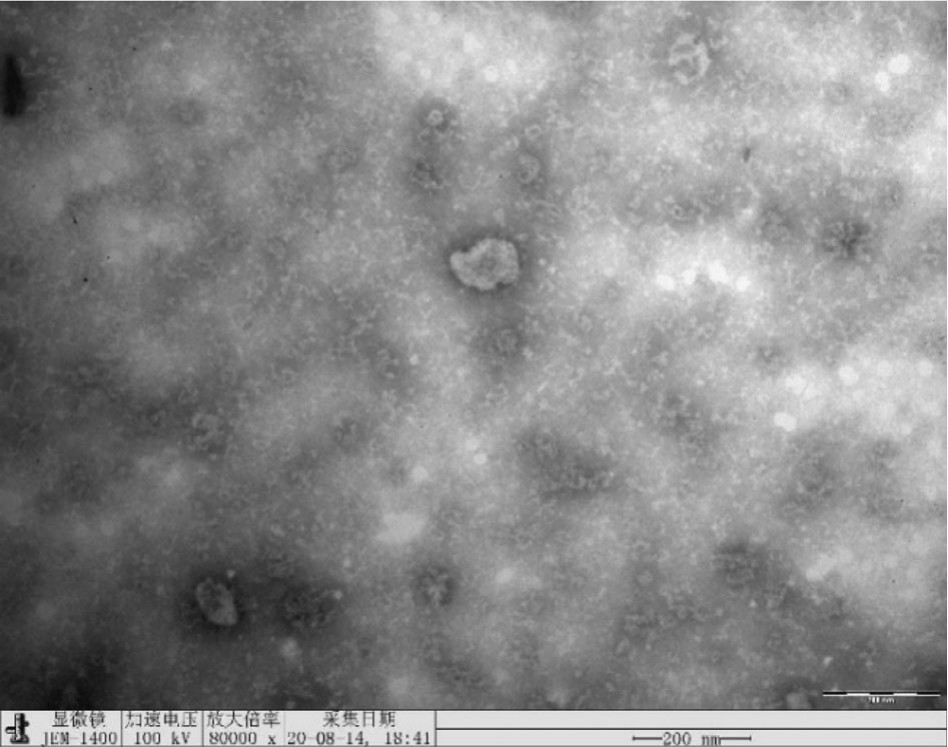

FIGURE C Electron microscopy results of the A549/PTX cell line correctly used in the current article(100 000x).

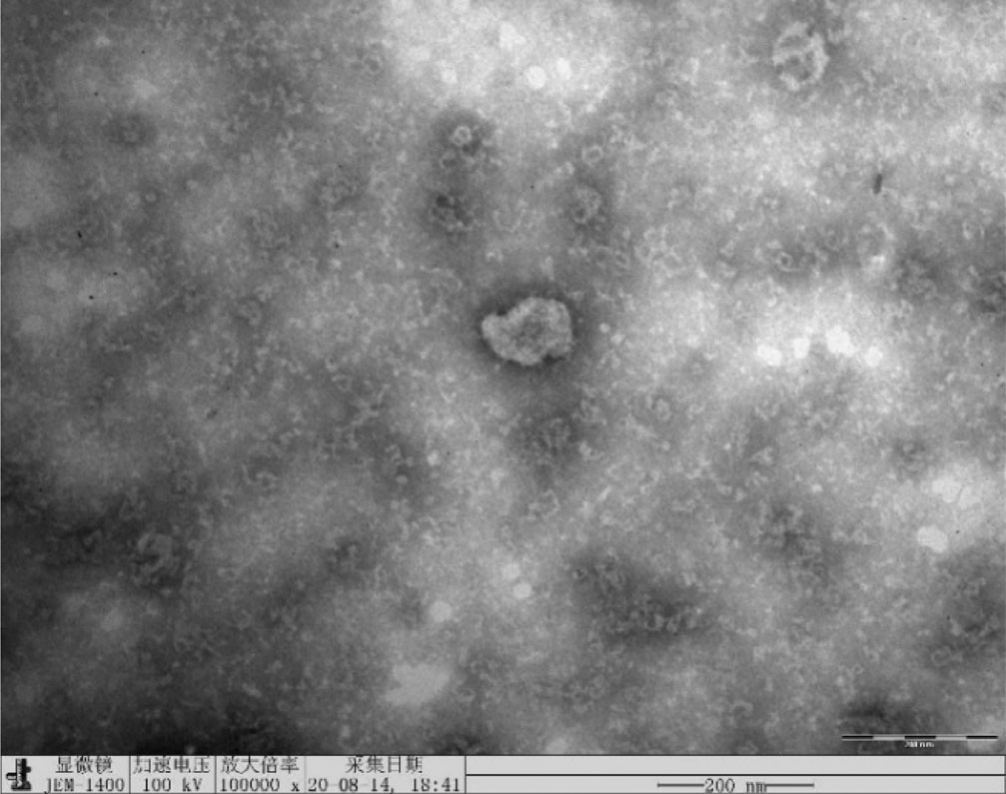

The authors apologize for the errors and any inconvenience they may have caused.

## References

[1] Li J , Zhu T , Weng Y , Cheng F , Sun Q , Yang K , et al. Exosomal circDNER enhances paclitaxel resistance and tumorigenicity of lung cancer via targeting miR‐139‐5p/ITGB8. Thorac Cancer. 2022;13:1381–90. 10.1111/1759-7714.14402 35396925 PMC9058310

